# Massive Pneumoperitoneum Presenting as an Incidental Finding

**DOI:** 10.7759/cureus.2787

**Published:** 2018-06-11

**Authors:** Harry Wang, Vivek Batra

**Affiliations:** 1 Internal Medicine, Thomas Jefferson University Hospitals, Philadelphia, USA; 2 Medical Oncology, Thomas Jefferson University Hospital, Philadelphia, USA

**Keywords:** spontaneous pneumoperitoneum, pneumoperitoneum, pancreatic cancer, incidental finding

## Abstract

Pneumoperitoneum is often associated with surgical complications or intra-abdominal sepsis. While commonly deemed a surgical emergency, pneumoperitoneum in a minority of cases does not involve a viscus perforation or require urgent surgical management; these cases of “spontaneous pneumoperitoneum” can stem from a variety of etiologies. We report a case of a 72-year-old African American male with a history of metastatic pancreatic adenocarcinoma who presented with new-onset abdominal distention and an incidentally discovered massive pneumoperitoneum with no clear source of perforation on surveillance imaging. His exam was non-peritonitic, so no surgical intervention was recommended. He was treated with bowel rest, intravenous antibiotics, and hydration. He had a relatively benign clinical course with preserved gastrointestinal function and had complete resolution of his pneumoperitoneum on imaging two months after discharge. This case highlights the importance of considering non-surgical causes of pneumoperitoneum, as well as conservative management, when approaching patients with otherwise benign abdominal exams.

## Introduction

Pneumoperitoneum is defined as the presence of free air within the peritoneal cavity. In the vast majority of cases (approximately 90%), this is a result of an intra-abdominal viscus perforation, often requiring intravenous antibiotics and acute surgical intervention [1]. Patients typically present with signs and symptoms of peritonitis and evidence of intraperitoneal free air on imaging (upright chest radiograph or computerized tomography (CT) scan).

However, there have been documented cases of pneumoperitoneum that do not involve viscus perforation [2]; this has been called “non-surgical” or spontaneous pneumoperitoneum (SP). Etiologies of this phenomenon are broad and can include intrathoracic, intra-abdominal, and gynecologic causes [3]. Patients with SP are often managed conservatively, and exploratory surgery is warranted only if the clinical features of peritonitis are present. Signs and symptoms of SP can range from asymptomatic incidental findings to an acute abdomen [4]; as such, it can become a significant treatment dilemma for physicians when the etiology is not immediately clear. We present a case of a massive pneumoperitoneum found incidentally in a patient undergoing routine surveillance imaging for pancreatic cancer. Written informed consent was obtained from the patient for publication of this case report and accompanying images.

## Case presentation

**Figure 1:**
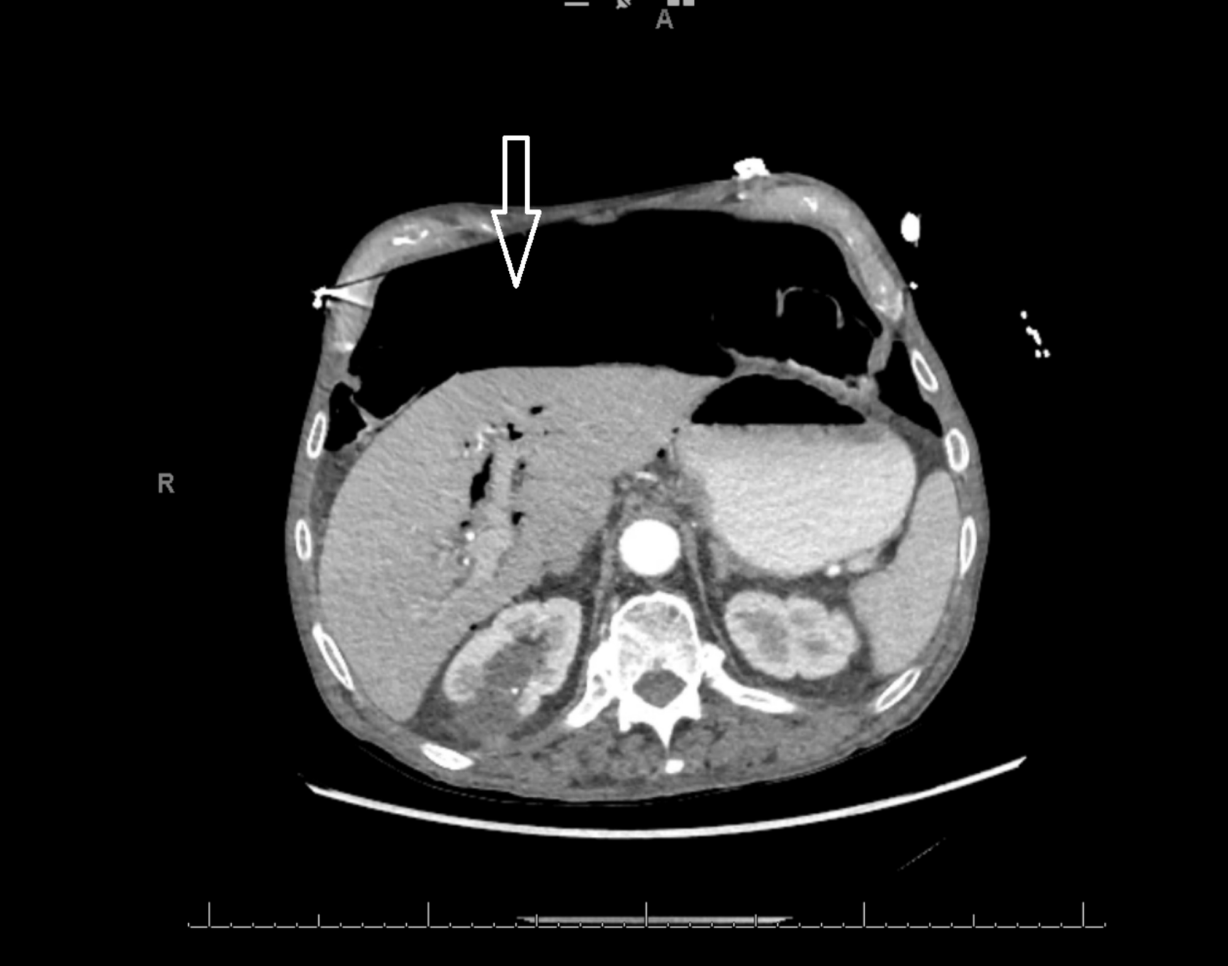
Axial CT scan of patient on admission showing free air in the peritoneal cavity (solid arrow) CT: computed tomography

**Figure 2:**
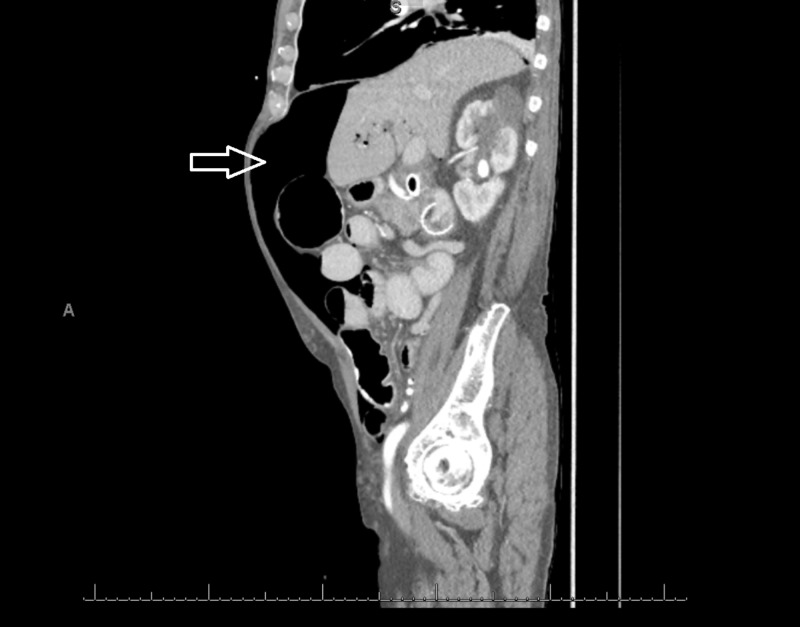
Sagittal CT scan of the patient on admission showing free air in the peritoneal cavity (solid arrow) CT: computed tomography

A 72-year-old African American male presented to the emergency department from the outpatient oncology office with a week of mild abdominal pain, which was not life-threatening. His past medical history was notable for metastatic pancreatic adenocarcinoma, which had been diagnosed one year earlier, from endoscopic retrograde cholangiopancreatography (ERCP) brushings when he presented with obstructive jaundice and required biliary and duodenal stent placement at the time. He had undergone a routine CT scan of his abdomen and pelvis for disease progression two days prior to admission, which revealed a massive pneumoperitoneum (Figures 1, 2). The ominous imaging prompted his admission to the hospital. On presentation to the emergency department, he was in no distress with a blood pressure of 126/85 mm Hg, pulse rate of 93 beats per minute, respiration rate of 18 breaths per minute, and a temperature of 36.9° C. Physical examination was significant for marked abdominal distension with a benign non-peritonitic exam and mild tenderness on palpation. Laboratory tests were unremarkable, except for a white blood cell count of 10.5 x 10^3^/uL with neutrophil predominance (83%). A repeat CT scan showed patent biliary and duodenal stents without a definite source of perforation; however, three suspicious locations were noted by the radiologist containing small foci of free air adjacent to the bowel.

**Figure 3:**
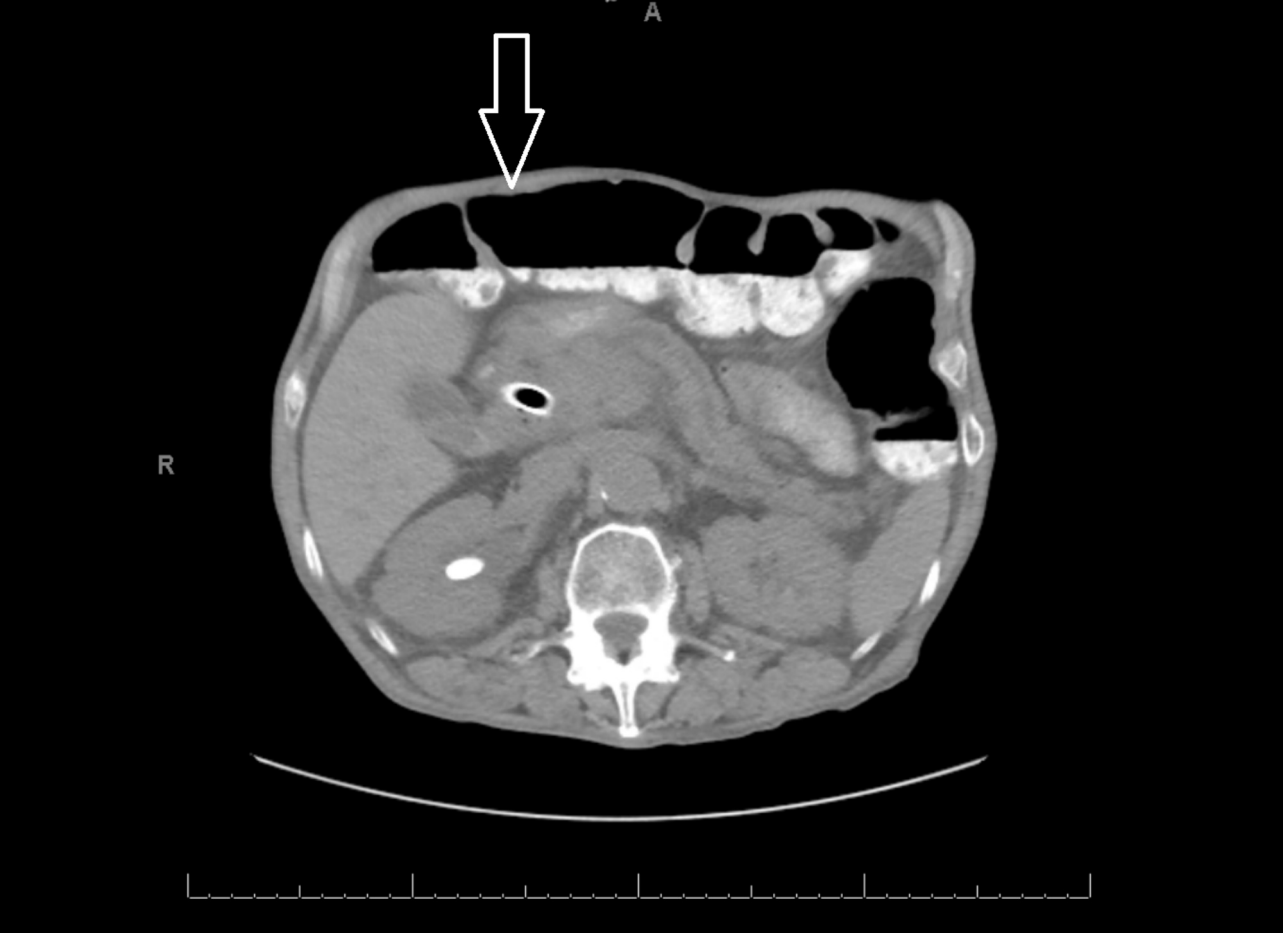
Axial CT scan two months after discharge showing resolution of the pneumoperitoneum (solid arrow) CT: computed tomography

Given the initial concern for perforated viscus, the patient was started on broad-spectrum antibiotics and antifungals. He was managed conservatively with intravenous hydration and strict bowel rest. He remained hemodynamically stable and non-septic throughout his hospitalization and continued to have flatus and intact gastrointestinal function. General surgery recommended no acute surgical intervention. In a few days, he was started on a clear liquid diet which he tolerated well. His abdominal distension gradually improved without any surgical intervention, and he was discharged home a week after admission. Two months later, he underwent a repeat CT scan that showed resolution of the pneumoperitoneum (Figure 3).

## Discussion

Despite its rare occurrence, spontaneous pneumoperitoneum can be attributed to a variety of causes. These can be roughly divided into four categories: intrathoracic, intra-abdominal, gynecologic, and idiopathic. Intrathoracic causes are among the more commonly reported cases and involve direct passage of air either through microscopic pleural and diaphragmatic defects or through retroperitoneal perivascular sheaths [2]. Intermittent positive-pressure ventilation, especially with high peak inspiratory pressures, can lead to air leakage into the abdominal cavity [5]; barotrauma causing pneumomediastinum, and subsequently pneumoperitoneum, has been described [6]. Other thoracic causes of SP that have been noted include asthma [7], chronic obstructive pulmonary disease (COPD) [8], cardiopulmonary resuscitation [9], and even severe coughing spells [3]. The most common intra-abdominal cause of SP is pneumatosis cystoides intestinalis, a rare condition characterized by the presence of submucosal and subserosal air-filled cysts throughout the gastrointestinal tract [10]. The pathophysiology is not well understood, as it can be associated with a range of gastrointestinal diseases, as well as pulmonary diseases and mechanical ventilation. As such, treatment is often supportive with supplemental oxygen, and most cases typically follow a benign course and resolve spontaneously. Pneumoperitoneum following abdominal surgery and endoscopy is another complication that, in most cases, is completely resorbed a few weeks after the procedure [3]. In women, free air can be transmitted through the genital tract to the peritoneal cavity under multiple conditions. Sexual intercourse, predominantly post-hysterectomy, has been reported to cause SP [11]. Vaginal douching, insufflation, and even pelvic inflammatory disease have been associated with non-surgical pneumoperitoneum [3]. Several bizarre incidences of SP have been documented after jacuzzi usage [12], scuba diving [13], and even knee-chest exercises [14]. Finally, there are cases of SP that demonstrate no clear risk factors or cause for the presence of intraperitoneal free air with subsequent negative laparotomies; these are labeled idiopathic pneumoperitoneum [15].

We report a case of a patient who presented with an incidentally found pneumoperitoneum on CT scan. Given his history of pancreatic cancer and stent placement, the concern for viscus perforation was high, as small bowel perforation from metastatic pancreatic cancer has been previously described [16]. Perforation is also a rare complication of ERCP, the incidence of which ranges from 0.3 - 1.3% [17]; however, this outcome typically occurs perioperatively, and our patient’s procedure and stent placement were performed about a year prior to the current admission. Further complicating the case was the presence of three reportedly “suspicious” locations on CT in which small foci of free air were found adjacent to the bowel. While no definite site of perforation was evident, there exists the possibility of subclinical micro-perforations, which would allow for the leakage of gas but not bowel contents through the viscera [2]. This mechanism has been proposed in some cases of idiopathic pneumoperitoneum, in which all other potential causes of SP have been ruled out. Ultimately, our patient did not require any surgical intervention, and his pneumoperitoneum resolved in several weeks with conservative management.  

## Conclusions

Spontaneous pneumoperitoneum is a rare but well-described phenomenon. A detailed history and physical examination with corroborative imaging studies are key to diagnosing this condition. The CT scan findings can appear worrisome; hence, it is necessary to monitor with serial abdominal exams and consult general surgery on presentation. Bowel rest, intravenous broad-spectrum antibiotics, electrolyte repletion, intravenous hydration, and serial abdominal exams are essential to managing this condition. Treatment is primarily non-surgical in the majority of cases, as long as the exam is non-peritonitic. Increased awareness about the various non-surgical causes may help to narrow the diagnosis and avoid unnecessary surgical interventions.
